# Efficient anomaly recognition using surveillance videos

**DOI:** 10.7717/peerj-cs.1117

**Published:** 2022-10-14

**Authors:** Gulshan Saleem, Usama Ijaz Bajwa, Rana Hammad Raza, Fayez Hussain Alqahtani, Amr Tolba, Feng Xia

**Affiliations:** 1 Department of Computer Science, COMSATS University Islamabad, Lahore Campus, Lahore, Pakistan; 2 Electronics and Power Engineering Department, Pakistan Navy Engineering College (PNEC), National University of Sciences and Technology (NUST), Karachi, Pakistan; 3 Software Engineering Department, College of Computer and Information Sciences, King Saud University, Riyadh, Saudi Arabia; 4 Computer Science Department, Community College, King Saud University, Riyadh, Saudi Arabia; 5 School of Engineering, IT and Physical Sciences, Federation University Australia, Ballarat, Australia

**Keywords:** Anomaly recognition, Crime detection, Video surveillance, Video analysis, Deep learning

## Abstract

Smart surveillance is a difficult task that is gaining popularity due to its direct link to human safety. Today, many indoor and outdoor surveillance systems are in use at public places and smart cities. Because these systems are expensive to deploy, these are out of reach for the vast majority of the public and private sectors. Due to the lack of a precise definition of an anomaly, automated surveillance is a challenging task, especially when large amounts of data, such as 24/7 CCTV footage, must be processed. When implementing such systems in real-time environments, the high computational resource requirements for automated surveillance becomes a major bottleneck. Another challenge is to recognize anomalies accurately as achieving high accuracy while reducing computational cost is more challenging. To address these challenge, this research is based on the developing a system that is both efficient and cost effective. Although 3D convolutional neural networks have proven to be accurate, they are prohibitively expensive for practical use, particularly in real-time surveillance. In this article, we present two contributions: a resource-efficient framework for anomaly recognition problems and two-class and multi-class anomaly recognition on spatially augmented surveillance videos. This research aims to address the problem of computation overhead while maintaining recognition accuracy. The proposed Temporal based Anomaly Recognizer (TAR) framework combines a partial shift strategy with a 2D convolutional architecture-based model, namely MobileNetV2. Extensive experiments were carried out to evaluate the model’s performance on the UCF Crime dataset, with MobileNetV2 as the baseline architecture; it achieved an accuracy of 88% which is 2.47% increased performance than available state-of-the-art. The proposed framework achieves 52.7% accuracy for multiclass anomaly recognition on the UCF Crime2Local dataset. The proposed model has been tested in real-time camera stream settings and can handle six streams simultaneously without the need for additional resources.

## Introduction

Surveillance systems continuously monitor ongoing activities in order to avoid or rescue any abnormal events. It is essential to improve the monitoring capability of such a system in order to ensure public safety (Piza et al., 2019). In recent years, a large number of CCTV cameras have been installed around the world to record ongoing activity or to serve as a record in the event of a mishap. Manual monitoring of such systems is costly, slow, human biased, and unreliable. Researchers are attempting to automate this process in order to facilitate security personnel and crime prevention authorities. Security surveillance based on computer vision is becoming more popular, and video-based activity recognition is one of them. A large amount of video data from CCTV cameras must be analysed in order to perform anomaly recognition. It is a difficult task because there is no clear distinction between normal and abnormal events because it is dependent on multiple factors and requires subjective definition. Other challenges include low-quality surveillance videos because CCTV typically captures events in low resolution, and most abnormal events occur at a distance. Abnormal events occur infrequently in nature whereas normal events involves huge variations, making annotation of each impossible. As a result of inability to provide adequate annotations and relying solely on one anomaly dataset may result in a high false positive rate. Another challenge is inter-class and intra-class variation problem as there is a thin boundary between normal and abnormal.

Initially, classical machine learning-based approaches attempted to solve these problems (Mehran, Oyama & Shah, 2009; Lu, Shi & Jia, 2013), but they proved impractical due to the cost of handling large amounts of data as well as computation time. Following the success of deep networks in various computer vision tasks, researchers presented a solution to perform anomaly detection. Currently, there are variety of 3D convolutional neural networks, such as deep neural networks, recurrent neural networks, and autoencoders, which are facilitating security surveillance system (Mansour et al., 2021; Ullah et al., 2021a; Ullah et al., 2019; Liu et al., 2018b; Sun et al., 2018). The few popular frameworks which have proven their worth are C3D (Tran et al., 2015), I3D (Carreira & Zisserman, 2017), ResNet 50 (Yao & Qian, 2018), and TSN (Wang et al., 2016). These algorithms are used to address the different domain challenges, such as intraclass and inter-class variation, where two or more classes have similar characteristics but are diverse. These methods incorporate multiple frames at a time for learning and therefore are better at discriminating classes that are difficult to distinguish using 2D convolution. Such approaches incorporate multiple frames per cycle during learning and forecasting and require a much larger amount of memory and computational resources (Canizo et al., 2019; Azizjon, Jumabek & Kim, 2020). Some researchers have tried to overcome this by reducing the input frame size and number of frames or by reducing the depth of the convolutional neural network (CNN) architectures but at the cost of accuracy. Ren et al. (2021), have highlighted opportunities and challenges of video anomaly detection and discussed various research directions. They have discussed some major issues, which includes ambiguity, dependency, privacy, noise, sparsity, and diversity.

**Figure 1:**
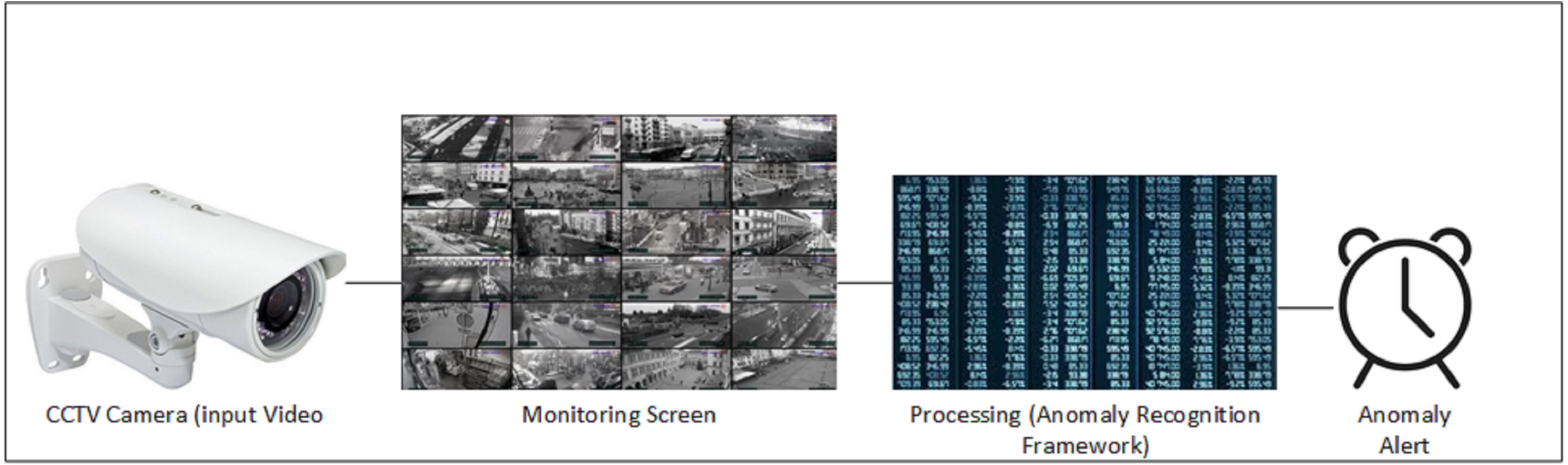
General data flow of anomaly detection framework.

Overall, majority of the systems are intended to achieve high accuracy on any specified dataset irrespective of computation cost. Recognition accuracy is important parameter to describe strength of a model but if accuracy comes at reduced cost then it becomes a practical solution. We proposed a 2D CNN based cost efficient anomaly recognition framework which is capable of extracting high dimensional data similar to 3D CNN models. It works through preprocessing the input data into frames, which produces normalized, resized, and augmented frames of the original data. These frames are then forwarded to the temporal feature extractor module, which extracts temporal behavior from the data through partial shift approach. Partial shift approach enables data exchange with neighboring frames over temporal dimension.It works by shifting learned features laterally with adjacent frames (*i.e.,* next, and previous frames). Temporal feature extractor is integrated into the residual block of the 2D CNN Baseline model so that Spatial features of input data can be extracted. We have used MobileNetV2 ResNet50 as the 2D backbone architecture in our experiment, which makes the anomaly detection problem more resource efficient. Figure 1 presents the general flow of our system using CCTV footage where Realtime camera stream is the basic source of information that is projected at all monitoring screens. Anomaly recognition model or system process that stream and detect anomalies whenever it occurred. This work used the UCF Crime dataset, which includes long, untrimmed videos with only video level annotation and no information on the temporal segment where the anomaly occurred. The available UCF Crime dataset involves few limitations, as discussed in Maqsood et al. (2021). This work attempts to overcome the highlighted issues through frame level data annotations and spatial augmentation, which improved the performance of video based anomaly recognition. This study aimed at improving spatiotemporal feature based learning as these features provide useful information to process anomalous videos. The following are the contributions of this article:

• We attempted to address the issue of the high resource requirement of the anomaly recognition method and proposed a lightweight, resource-efficient real-time streaming TAR framework that can be embedded on a simple machine like a central processing unit (CPU).
• We proposed to use temporal learning *via* partial shift operation to improve spatiotemporal feature based learning. It enables frames to share their learning among adjacent frames and reduce the cost of processing. Moreover, it helps in building feature maps based on high activity areas that support the classification task of anomaly recognition.
• Our framework is capable of performing online anomaly recognition and it allows six simultaneous screens on a CPU-based system while using fewer parameters (2.2M), FLOPs (0.564GFLOPs), model size (0.6Mb) and low latency overhead which proves it to be resource efficient approach.
• Our model achieves 7.87% and 2.47% increased state-of-the-art accuracy with ResNet-50 and MobileNetV2 respectively on UCF-Crime dataset.

The rest of this article is arranged as follows. In Section II, related work is discussed, and Section III represents the proposed anomaly recognition framework, where the method and dataset augmentation is explained. The experimental results and discussion are given in Section IV and the conclusion is given in Section V.

## Related Work

A lot of work has been done to perform anomaly detection tasks to support modern security surveillance systems, which include computer vision-based methods and deep learning-based frameworks. Most of these methods are focused on providing high anomaly recognition accuracy using 3D based deep networks which requires huge computation cost. Along with 3D networks, few researchers have worked on improving feature modeling to achieve higher accuracy. The majority of these methods provide reasonable accuracy, but computation cost and time are frequently overlooked, which is the primary focus of this study. We have discussed about some existing methods that are either based on 3D CNN models or describe feature modeling schemes. Both categories are related because we improved the spatiotemporal feature modelling and asserted that our proposed framework outperforms 3D CNN-based methods.

### 3D convolutional neural networks

3D convolutional neural network based approaches gained popularity due to their promising performance such as in Sultani, Chen & Shah (2018), where the authors have suggested a deep learning-based method for detecting anomalies in real-world surveillance footage. The authors proposed and developed a combination of two methods for detecting anomalies in a real-world video. Multiple instance learning (MIL), which automatically trains the model to construct a deep anomaly-ranking model that predicts high anomaly scores for anomalous video segments inside a video, is used to train their model.The primary contribution of the authors is the preparation of an anomaly dataset (UCF Crime) containing thirteen types of anomalous surveillance videos. Liu et al. (2018a) employed a Temporal Convolutional 3D Network (T-C3D), which is capable of real-time action recognition in videos because of its optimized network complexity and, as a result, substantially lower computing cost needs. Their framework for deploying T-C3D directs the network to learn video action representations in a hierarchical multi-granularity way. Maqsood et al. (2021) presented a framework for detecting real-world anomalies in video footage. They have proposed a straightforward but effective method for learning spatiotemporal features by training deep three-dimensional convolutional networks on the UCF Crime video dataset. Whereas, we repurposed a 2D CNN based architecture for our anomaly recognition framework which provides performance of 3D CNN.

### Feature modeling

#### Spatiotemporal modeling

Another approach is to improve spatiotemporal feature extraction process to perform anomaly recognition. Nawaratne et al. (2019) have targeted real-time anomaly detection through active/online learning and proposed the incremental spatial-temporal learner (ISTL), which can overcome the issues of both anomaly detection and localization. The proposed learner is evaluated by using a temporal threshold rather than focusing only on spatial threshold to save the contextual information from video feed. In 2019, Landi, Snoek & Cucchiara (2019) have worked by considering locality for anomaly detection of real-world videos. They have worked through enriching a considerable portion of an existing dataset with spatial and temporal domains with annotations, using spatiotemporal tubes instead of whole-frame video segments. Liu et al. (2020) have performed real time action representation using temporal encoding along with deep compression network. Their framework extracts video representations in a hierarchical granularity manner and use residual 3D CNN to extract appearance-based data. Similarly, Chu et al. (2018) have worked on anomaly detection problems by using results of sparse coding results of spatiotemporal features. Wu et al. (2020) have focused to improve the high feature extraction process and proposed a fast sparse coding network. Their method is based on the fusion of spatial–temporal features, and it achieves the highest performance at a maximum of 10,000 lower latency. Chang et al. (2022) have proposed an autoencoder architecture that is capable of learning spatial and temporal representations from videos. They have used the deep k-mean cluster to fuse the spatiotemporal features, which generate anomaly scores for anomalous events. Li et al. (2022) have worked on 3D integral images to perform anomaly detection and performed probability estimation using the Bayesian network. They have used a cube of a video as an event, which is represented as a histogram and then used the motion magnitudes and likelihood from the histogram template to estimate probabilities using prior knowledge.

#### Attention based modeling

Attention based strategies are also popular which involves extraction of high activity area to perform anomaly recognition. Ma & Zhang (2022) have proposed an attention-based framework using deep neural network-based architecture. The proposed framework includes an anomaly attention module (AAM) to score anomalies in a frame. Muhammad et al. (2022) have performed fine grained activity recognition to produce short video using video summarization. They have performed key frame extraction through deep CNN based attention modeling. Ullah et al. (2021a) have presented a timesaving deep feature-based intelligent anomaly detection framework for anomaly recognition. They have used CNN to extract spatial structure from a sequence of frame, which is then fed into a multi-layer bi-directional long short-term memory (BD-LSTM) model. Then, BD-LSTM can classify ongoing anomalous events in smart city surveillance setup. Duman & Erdem (2019) has worked on anomaly detection tasks through dense optic flow. The optic flow is calculated for input video to extract velocity and direction-based data, which is then passed to a convolutional autoencoder and then to Conv-LSTM encoder to detect anomalies. Ullah et al. (2021b) proposed an attention residual LSTM-based lightweight anomaly recognition framework. The authors proposed using a lightweight CNN architecture for feature extraction. They used the MobileNet architecture for frame wise feature extraction, preceded by a sequential learning strategy. Tang et al. (2020) have contributed to overcome the limitations of both reconstruction and future prediction methods. They claimed that their proposed framework is robust to noise and outperformed both baseline approaches used for reconstruction and prediction tasks. Zhong et al. (2022) have performed anomaly detection using a cascaded reconstruction model along with an optic flow network and used the reconstruction error to choose an anomaly detection framework. Dong, Zhang & Nie (2020) have proposed a semi-supervised learning-based framework that can predict future normal frames for a given feed. Their proposed framework works through using two discriminators and a generator to target motion information from videos *i.e.,* optic flow.

Most of the existing work is focused on increasing accuracy of anomaly recognition that usually based on deep networks with high computational requirements. Recently, researchers are considering cost effective anomaly recognition systems, which still requires many improvements. In particular, online anomaly recognition process long untrimmed sequences, which is a challenging task. Our study tries to reduce the computational cost of anomaly recognition process without compromising on accuracy. It processes online streams and resolves the problem of long untrimmed video. We have performed anomaly recognition which identifies whether there is anomaly in data or it is a normal sequence. We used partial shift strategy to incorporate spatiotemporal learning in our framework which improves the accuracy of 2D CNN based architecture to outperform 3D CNN based methods.

## Temporal Anomaly Recognizer (TAR)

### Overview

Deep learning-based solutions have outperformed many existing models, but the computational cost of such systems became another bottleneck. In real time scenarios, high resource systems are not useful which means cost of system is equally important as its efficiency. Anomaly recognition is a complex task and processes large amount of data such as 24/7 Real-time camera streams. We have considered both constraints: large amount of data and efficiency of system in terms of timely recognition. Therefore, this work attempts to provide an efficient anomaly recognition framework, which addresses the problem of high resource requirement while working with deep learning based methods. We have used 2D Convolution-based architectures due to their outstanding performance in a wide range of domains. 2D Convolutional networks are good for low-level feature extraction, but they are ineffective for capturing high-level information, such as temporal information (Lin, Gan & Han, 2019). As compared to 2D CNN models, 3D CNN models are capable of extracting high dimensional data but at very high cost especially when we have to process the video stream. This study provides a method to perform 3D computations at cost of 2D model through using data shift operation. For example, videos are multidimensional array (batch size, number of channels, temporal, and spatial points) which requires expensive computations as compare to image. As each video is first converted into frames during prepossessing stage and these frames are representation of source video. 2D CNN avoids temporal dimension during learning process. In contrast, our temporal feature extractor (TFE) performs temporal modelling through shifting channels in both forward and backward direction so that information within frames can be exchanged. The process of shifting channels is explained Temporal feature extractor section. To perform anomaly recognition, we need a model which can extract spatial features of data too so we need a 2D CNN base model for example, MobileNetV2. We propose a temporal anomaly recognizer (TAR) in which we used a partial shift strategy in our method to transfer temporal learning across ConvNet layers. The process of using temporal learning and spatial data is explained in section Anomaly Recognition. So, Temporal Anomaly recognizer which is our proposed framework, is described in terms of of temporal feature extractor and a backbone 2D CNN model.

**Figure 2:**
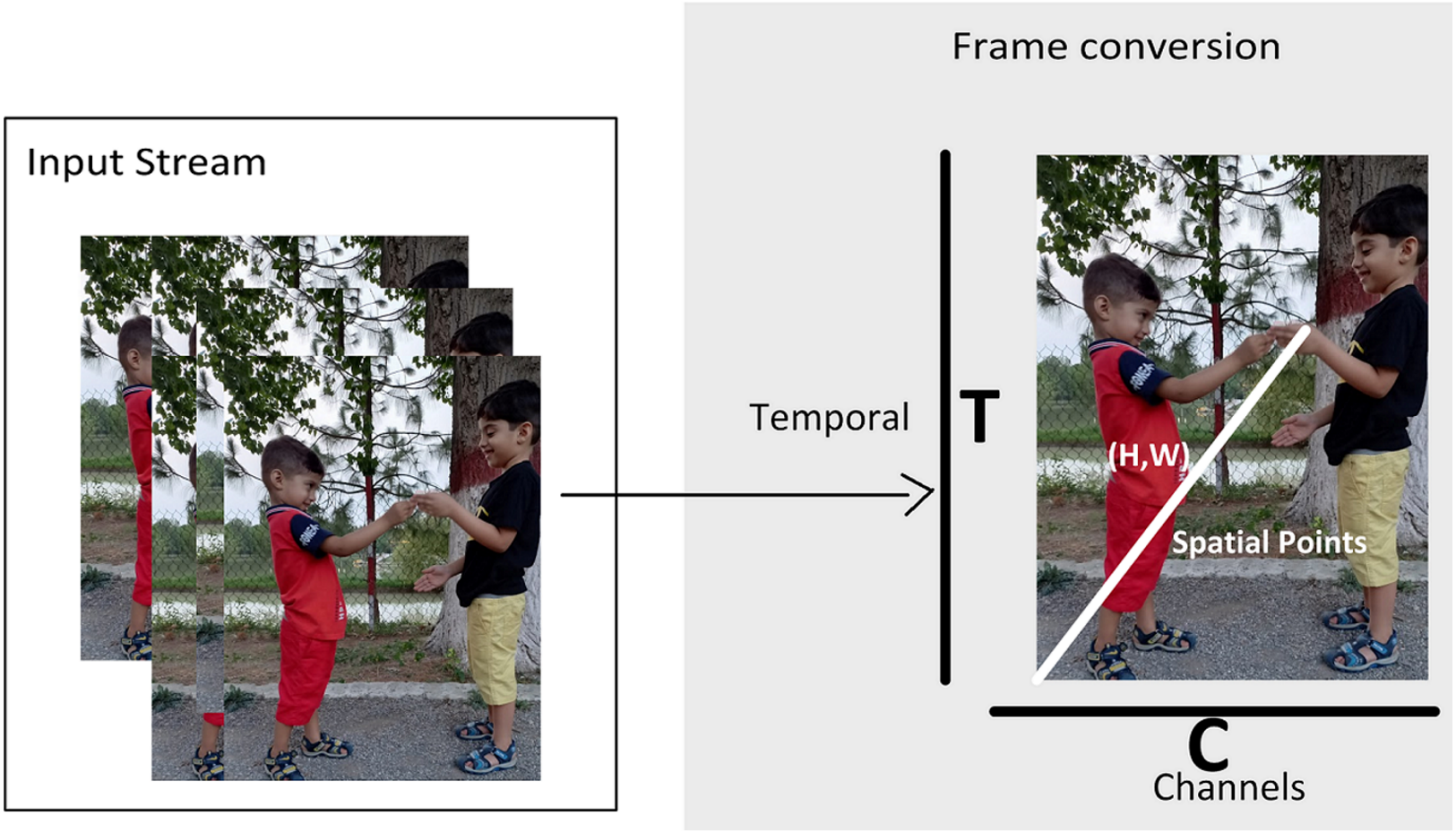
Video representation in terms of multidimensional array.

**Figure 3:**
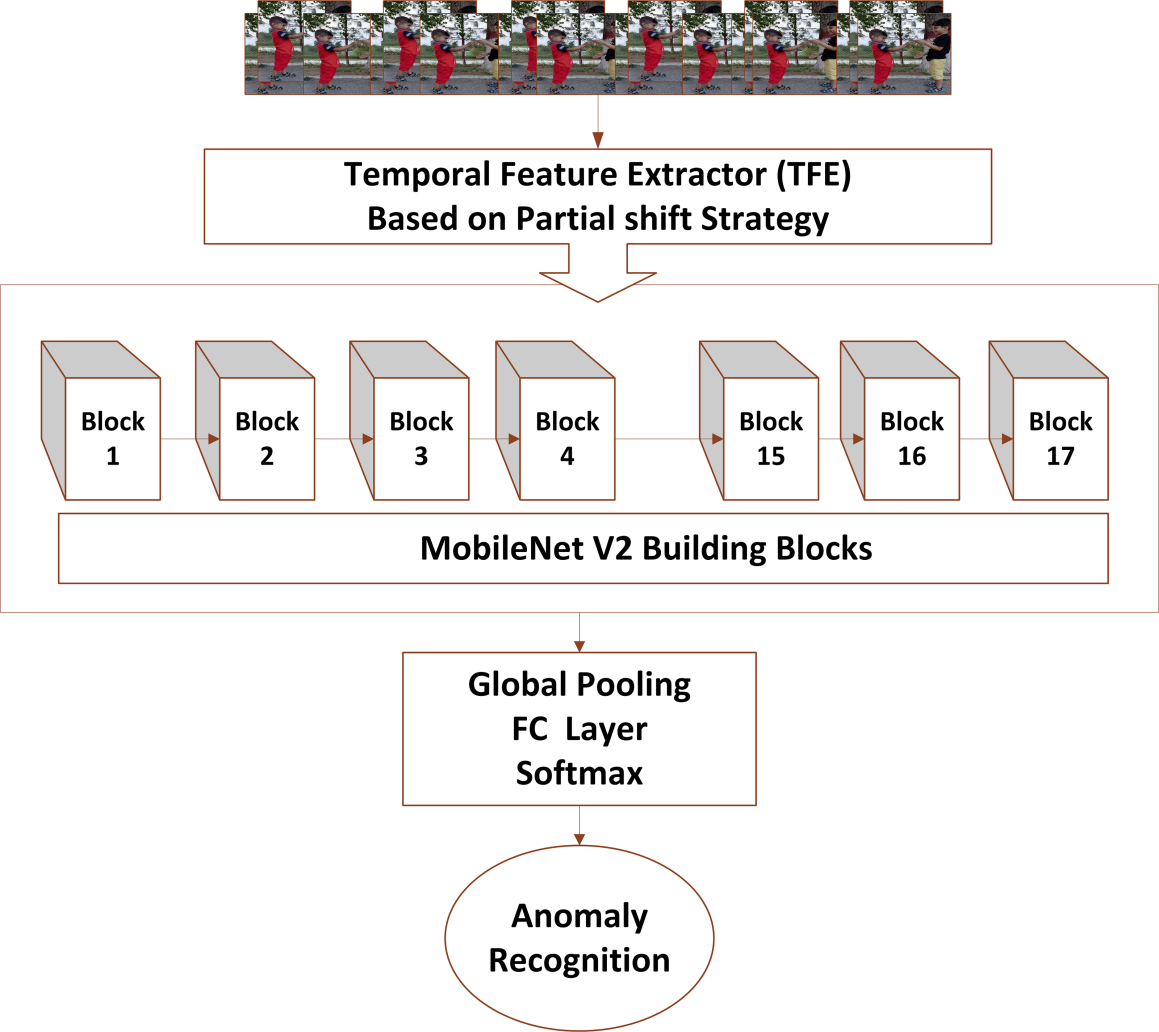
Video input is forwarded to temporal feature extractor which performs temporal and spatial modeling with the help of MobileNetV2 whereas fully connected layers perform anomaly recognition *via* spatiotemporal modelling.

**Figure 4:**
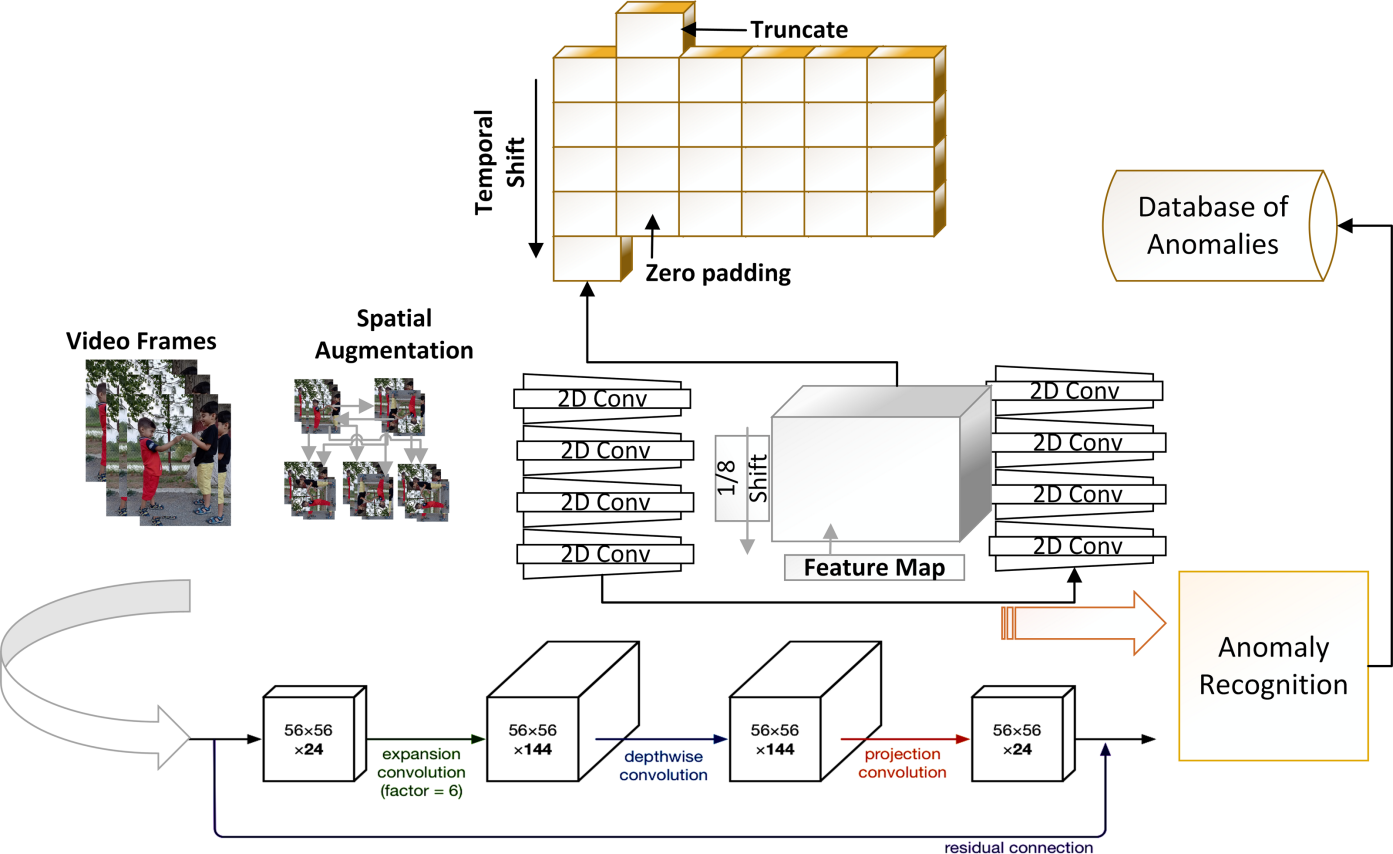
Proposed temporal based anomaly recognizer (TAR) framework with 2D MobileNetV2 baseline architecture.

Figure 2 shows the multidimensional tensor of a video which needs to be processed during anomaly recognition process. Video activations can be termed as N Batch size, C number of channels, T temporal and H,W spatial points. TAR has three main components, which includes preprocessing of data to get normalized and augmented data for training. The other part is temporal feature extractor, which serves the purpose of reducing model size and number of parameters with high speed performance so that our framework can be used in practical scenarios. The third component performs anomaly classification using spatial features of the data and temporal learning of second component. Figure 3 depicts the temporal feature extractor and Fig. 4 the proposed framework (TAR), which is built on top of the residual connection of MobileNetV2. We have experimented with two different models MobileNetV2 and ResNet 50 to observe the performance variation to achieve a computationally cost-effective solution whereas MobileNetV2 is our final backbone model.

### Preprocessing

The first step is to divide the video into frames, and data values are scaled by mapping data pixels between 0 and 1. The next step is to resize data frames, and we choose 170x170 after considering other higher resolution scales. We chose this as the final value because there is no significant change in system performance on higher scales. Prior to applying the augmentation technique, the frames are normalized. Data augmentation is a popular approach that is used to increase data examples of a scene without exploiting its meaning. As videos are represented by the sequence of frames which are like image domain problems in various characteristics. Image based methods work within the spatial domain since there is no temporal dimension so linear transformations are the common method to perform data augmentation in such scenarios. Whereas video data augmentation is complex and prone to noise. Such as without considering the temporal dimension, any transformation within data can change the entire meaning of the video. The task of video data augmentation can be simplified if video annotations are available which is another open challenge (Um et al., 2017; Cui, Goel & Kingsbury, 2015). In this study, we used frame-level annotations of data which reduces the complexity of the task and we applied spatial augmentation to our data to increase data example. Our data is first converted into a fixed number of frames, which were then horizontally and vertically flipped. Such scenarios where data is already prone to noise (*i.e.,* anomalies) both horizontal and vertical flips are preferable for data augmentation (Maqsood et al., 2021). After performing spatial augmentation, frames are converted to create a video after performing vertical and horizontal flips.

### Temporal feature extractor

A video is multidimensional array and expensive to process as compared to 2D images. For 2D image problems, we have plenty of algorithms such as VGG 16, ResNet 101 and CNN. However, this study is based on anomaly recognition from videos to provide security surveillance which requires both spatial and temporal modelling. After preprocessing of data, next step is feature extraction and temporal feature extractor is part of TAR. The intuition behind the method is to use 2D CNN along with a partial shift strategy, which can learn and pass temporal information over the frames. In Fig. 2 T is giving frames and C Channels which are being processed. Convolutional neural network models are built on ConvNet layers and mathematical expression of 2D Convolution is shown in (1):

(1) \begin{eqnarray*}y[a,b]=\sum _{m=-\infty }^{\infty }\sum _{n=-\infty }^{\infty }h[m,n].x[a-m,b-n].\end{eqnarray*}

Here h is the kernel matrix with m and n indices whereas is the input image matrix with *a* and *b* indices. For input vector *A* and weight vector *W*, convolution (*X* = *Conv*(*W*, *A*)) requires weights (*w*1, *w*2, *w*3) which is convolved with input vector *A*. Hence, Convolution value is as: *X*_*i*_ = *wA*_*i*−1_ + *wA*_*i*_ + *wA*_*i*+1_. Rather than applying convolution by shifting and multiply-accumulate operations, the input channel has shifted as −1 and +1. The shift operation will transfer the information with its neighbor frames (*i.e.,* (*A*_*i*−1_, *A*_*i*_, *A*_*i*+1_). The shift operation is shown in Fig. 3 and Eqs. (2), (3) and (4):

(2) \begin{eqnarray*}{A}_{i}^{-1}={A}_{i-1}\end{eqnarray*}

(3) \begin{eqnarray*}{A}_{i}^{0}={A}_{i}\end{eqnarray*}

(4) \begin{eqnarray*}{A}_{i}^{+1}={A}_{i-1}.\end{eqnarray*}

Hence, we can use shift and multiply-accumulate operations in such a way that it will reduce computation cost. Computation cost of a model is basically number of parameters of a model which need to be processed to perform a task such as anomaly recognition. By considering a 16 frame input sequence, we have a lot of features which needs to be extracted and so multiple channels over temporal dimension. If we do not shift any channels within frames like 2D CNN then we cannot extract temporal behaviour of data. As shifting of channels cause the exchange of temporal learning which is our ultimately goal to extract from data. One solution is to shift all the channels so that we can have maximum temporal learning and eventually our model can achieve a better performance. Shifting channels across the temporal dimension may involves huge data movement which means high cost model and drawback of a system. However, if we partially shift our channels (*i.e.,* 1/8) then it will provide temporal learning as well as reduced cost.

Hence temporal feature extractor shifts channels in both direction +1 in one direction and −1 in other to exchange data in neighbourhood frames. This allows us to extract high-dimensional data features (temporal data), which is impossible with 2D CNN. Along with temporal features, spatial features are also important because temporal features describe changes over time, whereas spatial features extract interactions within a frame. Shifting channels can result in data loss because the information from the current frame is no longer available, resulting in reduced spatial features. The shift operation is associated with the convNet layers of the 2D CNN model, and it is necessary to understand how the temporal feature extractor (TFE) collaborates with 2D CNN to perform anomaly detection. Consider the convNet layers of MobileNetV2 which means shifting can be done before convNet layers (outside of residual connections) or within residual connections. Using channel shift outside of the residual branch may result in spatial data loss because we cannot access the original data of the frame. However, if we apply shift within the residual branch, we can still access the original data of a frame, indicating that spatial data is not jeopardized. The 2D CNN model aids in the extraction of spatial features, while partial shift strategy aids in the extraction of temporal features.

### Anomaly recognition

Temporal and spatial modeling provides temporal and spatial behaviour of data which is then forwarded to perform recognition using a classification algorithm. The method used to perform anomaly recognition is based on temporal feature extractor module and 2D convolutional architecture *e.g.*, ResNet-50 and MobileNetV2. The framework takes video as input that is converted into frames and then data augmentation modules perform spatial augmentation of data. Spatial augmentation produces new training examples to increase the amount of labeled data. Then, the temporal feature extractor learns and transfers the temporal information of each frame to its neighborhood frames. The temporal feature extractor used the strategy as discussed under temporal feature extractor heading. As shown in Fig. 4, after performing preprocessing and data augmentation, our model performs feature extraction to extract useful data from anomalous videos. In Fig. 4, a tensor is shown which shows channel shift in both direction along temporal dimension. This represents Deep features are extracted through a combination of MobileNetV2 model and partial shift strategy.

Within the architecture of MobileNetV2, there are two type of blocks: residual blocks and downsizing blocks. The stride of a residual block is one, but the stride of a downsizing block is two. Each block comprises three layers: a convolutional layer with a non-linear cost function, a depth wise convolution layer, and a convolutional layer without a non-linear cost function. We proposed using a partial shift function within MobileNetV2′s residual blocks to extract both spatial and temporal data. To lower the model’s latency overhead, the model stores only 1/8 of the feature set for an incoming frame to reduce the computation cost of the framework. The partial shift function changes the information in the current frame along temporal dimensions and exchanges the learned parameters with associated or neighboring frames. The next component is anomaly identification, which uses features to divide data into anomaly and normal classes. The final feature map, which is used by the classification layer, is improved further by applying a variance-based filter, which analyzes the variance of extracted features and highlights those with high variations. This method will improve the final feature map, which will be utilised to identify anomalies during the classification stage. Anomaly events are saved in an anomaly database after they have been identified based on recognition results. The identified anomalous events are saved using hardware based implementation as we have tested our method by designing a surveillance application using Jetson Nano. However, our proposed framework is limited to perform anomaly recognition task only.

### MobileNetV2 architecture

For anomaly recognition, we propose using both temporal and spatial features. As a result, MobileNetV2 is used for spatial feature extraction and anomaly classification (*i.e.,* anomaly, normal). MobileNetV2 is a popular convolutional-based architecture that is specifically designed to operate on low-resource systems. The MobileNetV2 has the same residual block as the V1, but it also has an expansion layer, projection layer, and depth wise convolution layer. The bottlenecks residual block is a MobileNetV2 building block that shrinks the input to reduce computation. Our framework is built on top of MobileNetV2, which provides lower computation costs. Temporal feature extractors are useful for transferring temporal learning, but spatial learning of video frames is also important and should be saved for prediction. The bottleneck residual block’s function is to extract spatial features from video frames. As a result, the model can extract both spatial and temporal features from video data while maintaining a reasonable recognition accuracy.

## Experiment

### Dataset description

The objective of anomaly detection from surveillance videos is to find data points or elements that do not meet the criteria of a normal class. We require data that can depict the problem at hand during the model evaluation phase. There are many anomaly-based datasets available; however, the most of them are actor-based, which are valuable for experiments but limited in their ability to capture real-time variations of a scene. For this study, we have used UCF Crime dataset (Sultani, Chen & Shah, 2018) and UCFCrime2local dataset (Landi, Snoek & Cucchiara, 2019), which are based on real-time surveillance videos.

UCF Crime (Sultani, Chen & Shah, 2018) is a large scale video dataset of untrimmed videos, which means an anomalous video may have few frames which are part of anomaly. It is based on 13 real-time anomalies, which are arson, assault, fight, robbery, burglary, accident, vandalism, explosion, abuse, arrest, shoplifting, shooting, and stealing. The last and 14th class contains normal events only. The dataset offers few limitations due to which the performance of a system can be compromised, such as anomalous video may have more normal frames than the anomaly frames. This issue can cause model biases over normal events. The other issue is an intra-class variation which may cause over fitting of the model (Maqsood et al., 2021). UCF Crime2Local (Landi, Snoek & Cucchiara, 2019) is the subpart of UCF Crime dataset and contains six anomalous classes which are burglary, robbery, assault, vandalism, arrest, and stealing. The UCF Crime2Local provides the temporal annotations for anomalies that are missing in UCF Crime.

### Data processing

UCF Crime only provides annotations at the video frame level, which means that each video frame has the same label. We have spatially annotated video data, which means that each frame has relevant annotation. This process also improves training by providing enough labelled data and a training set based on anomalous frames. The dataset necessitates frame-level annotations and has a class imbalance issue. To perform data augmentation and resolve class imbalance issues, this study employs a preprocessing strategy as discussed in previous Preprocessing section. We require a large amount of data for model training, which has been generated using data augmentation strategies. Data augmentation generates new data samples with correct labels as well as view invariants of the same frame (*i.e.,* image). This study employs spatial augmentation techniques to perform data augmentation, as well as frame level data annotation. Each video is divided into frames, which are then used to perform vertical and horizontal flips for data augmentation.

### Training and testing

The training split of the UCF Crime dataset has been expanded to include more training examples, while the testing split remains the same as provided by CRCV. The testing split consists of 168 temporally annotated anomalous and normal videos, whereas our augmented training split consists of 1120 (560 normal, 560 abnormal) videos. On Colab, we trained the deep neural network with a Tesla T4/K80 GPU. The temporal feature extractor operates in conjunction with two different 2D CNN baseline models, Resnet50 and MobileNetV2. The training strategy included disk-resident frame extraction with a python-based directory/file parser and OpenCV for automated dataset labelling (normal *vs.* abnormal).

To achieve the desired output, the training phase of the anomaly recognition framework includes data handling and hyperparameter tuning as given in Table 1. The video frames from the UCF Crime dataset are used, with training epochs set to 100 and batch size set to 16. Batch normalization is carried out using stochastic gradient descent, with the model’s learning rate set to 0.01, and dropout set to 0.5 with a decay of 0.1 after 50 epochs.To improve the feature extraction process, we used a MobileNetV2 model that had been pretrained on the imageNet dataset. The same setup is used to experiment with ResNet50 on the UCF Crime dataset. Experiments are carried out on PyTorch platforms and data modeling libraries.

## Results

### Comparison with state-of-the-art methods

In this article, we repurposed a 2D CNN model to extract high-dimensional data such as temporal information, which is important in video-based anomaly recognition. In the preceding section, we thoroughly discussed the proposed TAR and our experimental setup. We have compared its performance to other state-of-the-art methods, such as Ullah et al. (2021b) have used an attention residual LSTM-based lightweight anomaly recognition framework. They proposed using a lightweight CNN architecture for feature extraction. The extracted features have fed into a residual attention LSTM, which has trained to learn contextual information in abnormal activities. Moreover, the authors claimed that residual attention LSTM is 10% more efficient in terms of learnable parameters than conventional LSTM. Ullah et al. (2021a) have worked on a time saving deep feature-based intelligent anomaly detection framework for anomaly recognition. They have extracted spatial structure from a sequence of frames using a pre-trained convolutional neural network (CNN) model. Then, BD-LSTM can classify ongoing anomalous events in smart city surveillance setup. The authors have experimented with their proposed framework on UCF Crime and the UCF Crime2Local dataset. Biradar, Dube & Vipparthi (2018) have proposed DEARESt for aberrant behavior detection in surveillance videos. DEARESt employs a two-stream network to extract appearance and motion flow features separately, from a video stream. Then, these features are concatenated to form a single feature vector that is further used to classify a video.

**Table 1:** Hyperparameters settings of experiment.

Hyperparameter	Hyperparameter value
Batch size	16
Epochs	100
Learning rate	0.01 (decays by 0.1 at epoch 40 80)
Dropout	0.5

Convolutional neural network based models are getting popular in computer vision applications. Most of the systems are based on deep networks, which made them resource dependent. This study proposed to use resource efficient strategy without compromising on accuracy of the system. MobileNetV2 and ResNet-50 are used as the experiments’ backbone 2D convolutional architecture. However, due to its resource efficiency, the MobileNetV2 architecture is considered the final model for the proposed study. MobileNetV2 is used for spatial feature extraction and in conjunction with a temporal feature extractor (TFE) for temporal modeling. We conducted experiments on two benchmark datasets, UCF Crime and UCF Crime2Local. The study compared the performance of UCF Crime’s testing split to that of existing methods. During experiments, we also used ResNet-50 to investigate differences in accuracy and resource utilization.

**Figure 5:**
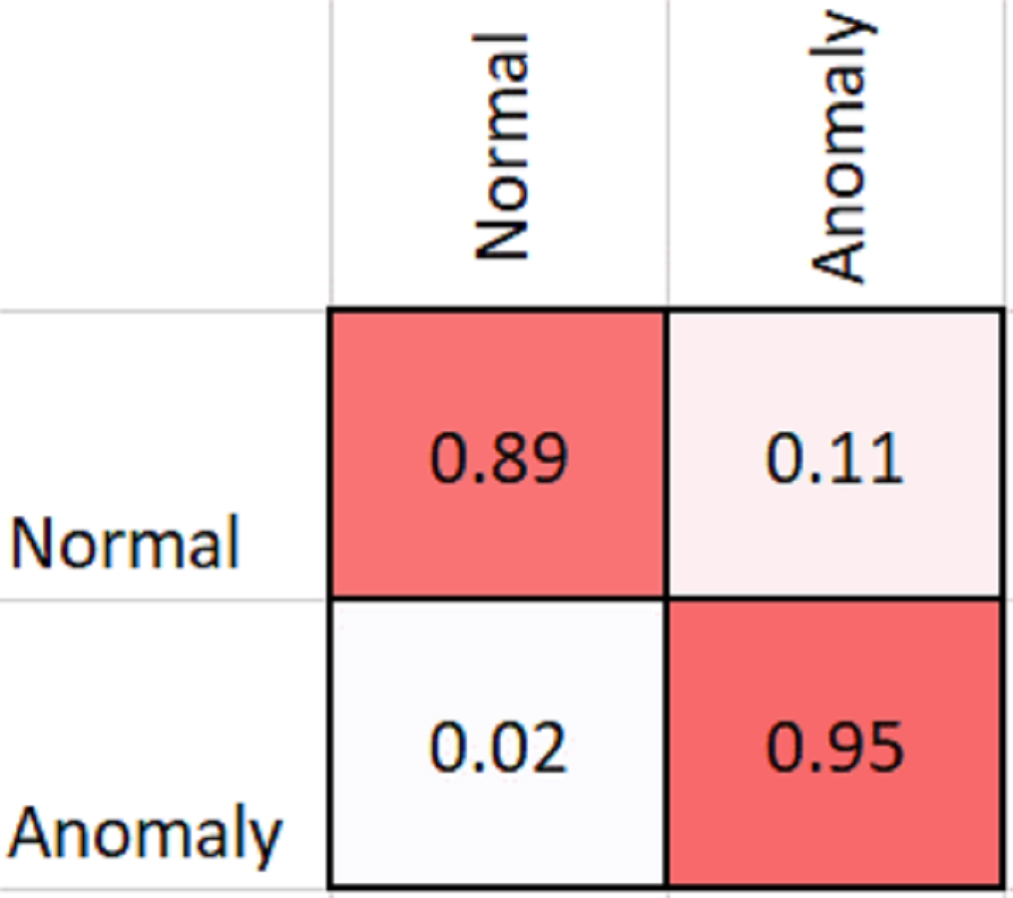
Confusion matrix of proposed framework with ResNet50 as the 2D CNN baseline model.

**Figure 6:**
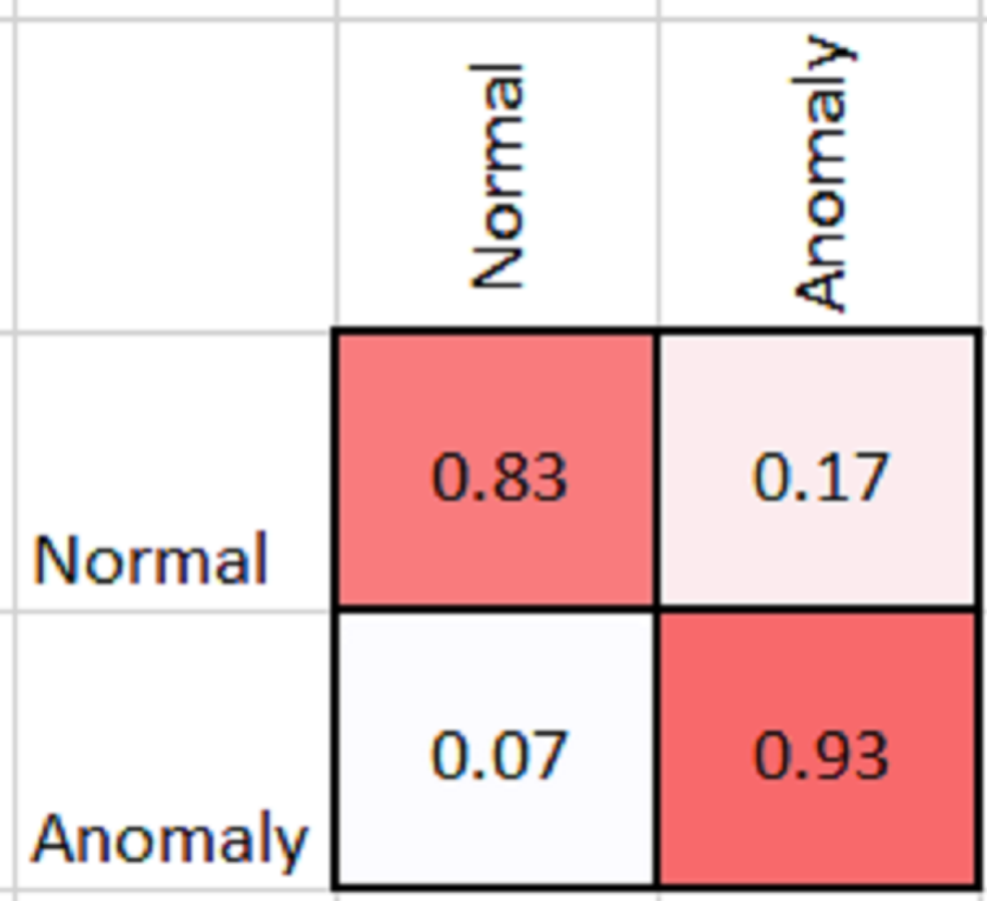
Confusion matrix of proposed framework with MobileNetV2 as 2D CNN baseline model.

**Figure 7:**
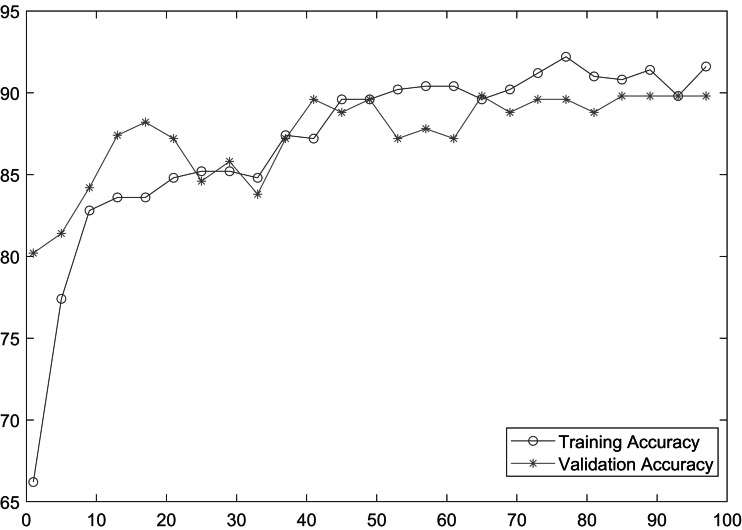
Accuracy of proposed framework.

**Figure 8:**
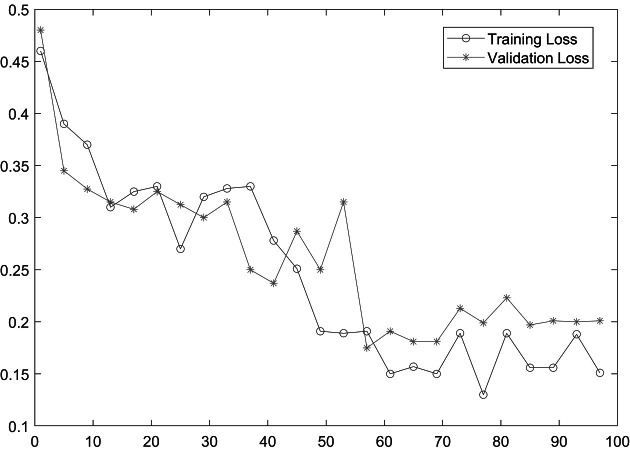
Loss curve of proposed framework.

As shown in Table 2, MobileNetV2 outperforms in terms of resource utilization with compromising only a minor amount of accuracy. Table 2 includes different measures to compare the performance of our model with other methods, such as accuracy, precision, recall, model parameters, model size, and FLOPs of the model. We trained the proposed framework with two different architectures *i.e.,* ResNet50 and MobileNetV2. The MobileNetV2 provides accuracy of 88% with a loss of 0.201 and ResNet provides accuracy of 93.4% with a loss of 1.2101. We have also drawn a comparison of high resource system and low resource system based performance. In Figs. 5 and 6, we have presented confusion matrix of proposed framework with both ResNet50 and MobileNetV2 models. Confusion matrix describes classification behaviour of a model such as in terms of true positive (TPR) and false positive rate (FPR). As shown in the figures, ResNet50 achieves better performance in terms of recognition rate as compared to MobileNetV2 but we selected MobileNetV2 as final baseline architecture as our agenda is to provide resource efficient framework. Figs. 7 and 8 shows accuracy and loss curve of proposed anomaly recognition framework on UCF crime dataset with MobileNetV2 as baseline model. We have implemented our framework using NVIDIA Jetson Nano to compare performance for edge devices with CPU and GPU. Moreover, our method provides fast computation speed with time complexity of 0.198 s. While discussing performance of our method, its resource efficiency is also notable. There is a difference in the number of frames processed per second, so GPU based systems are fast in prediction. CPU based systems usually have slow processing speed, but it does not affect the recognition rate. The prevalence of CPU-based systems in real-time scenarios is the primary motivation for proposing a resource-efficient system. Our model work efficiently on both CPU and GPU and produces low latency of 42.1 ms and 12.01 ms, respectively. That is why, we have claimed that our proposed framework (TAR) is resource efficient.

**Table 2:** Comparison of the results achieved using ResNet50 and MobileNetV2 architecture.

**Method**	**Accuracy**	**Precision**	**Recall**	**Model size (MBs)**	**Parameters**	**Mega FLOPs**
Attention Residual LSTM (Ullah et al., 2021b)	78.43%	87%	78%	12.8	3.3M	618.3
DEARESt (Biradar, Dube & Vipparthi, 2018)	76.786%	–	–	1187.5	305M	–
ResNet50+multi-layer BD-LSTM (Ullah et al., 2021a)	85.53%	–	–	143	25M	–
TAR(Baseline ResNet50)	93.4%	97.8%	89%	91.2	23.5M	6768
TAR(Baseline MobileNetV2)	88%	92.2%	83%	8.61	2.2M	564

**Figure 9:**
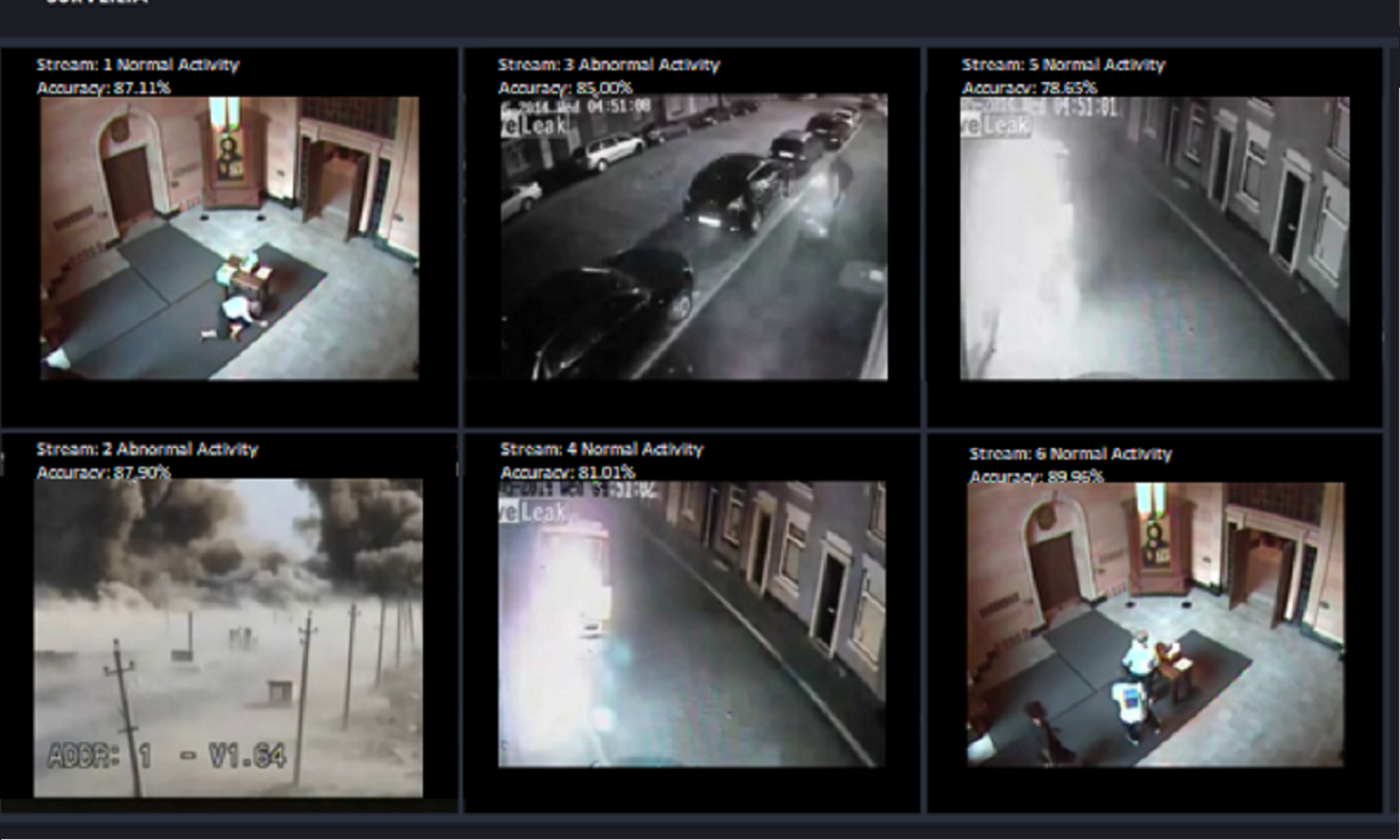
Performance of our desktop application based on proposed framework (TAR).

**Figure 10:**
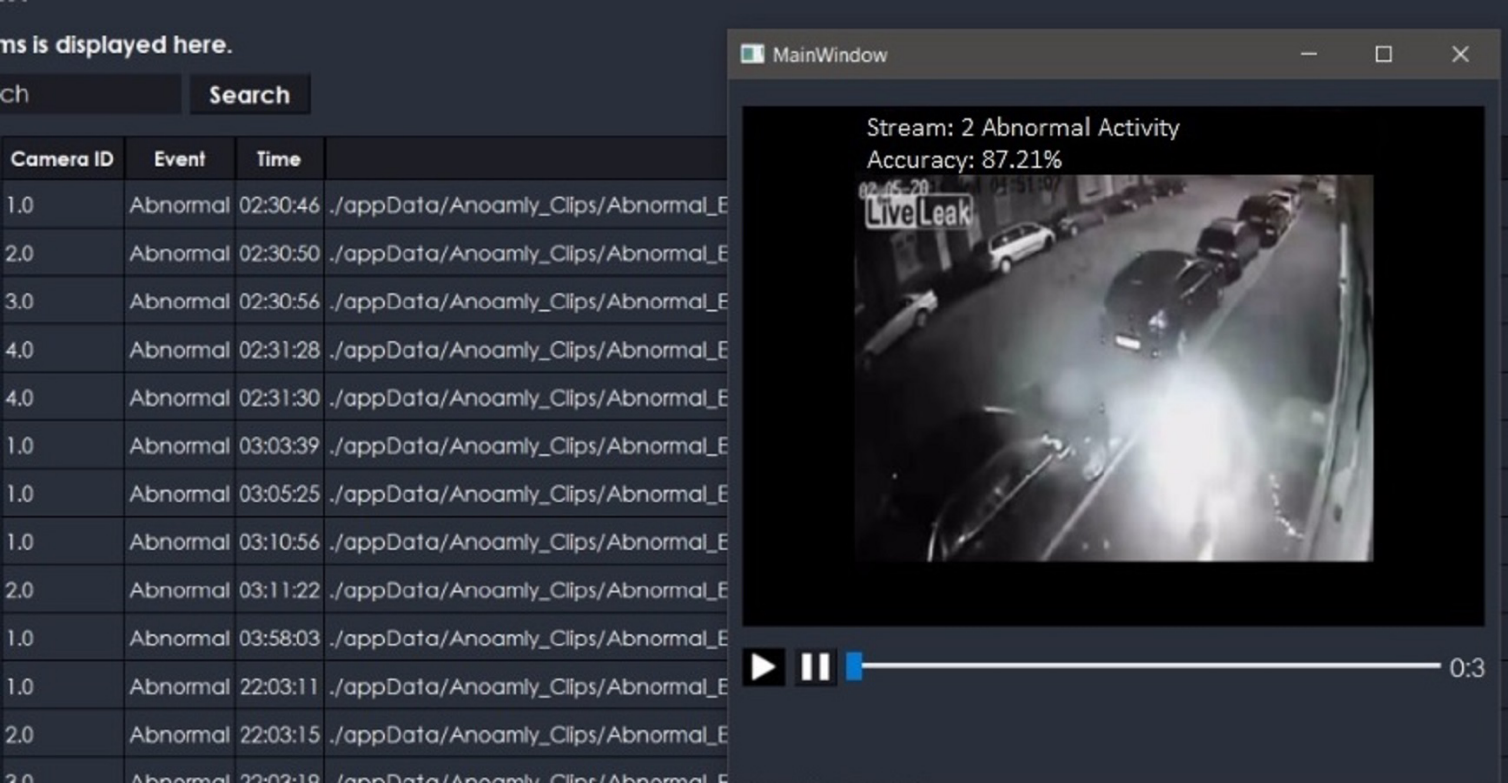
Desktop application stores anomaly clips.

The proposed system is also extended for a desktop application to serve the purpose of security surveillance as presented in Fig. 9. It has functionality to store the online streams as a database, which stores only anomalous frames from camera streams as shown in Fig. 10. The purpose of managing a database of anomalies is twofold, *i.e.,* it can be used for the forensic purpose to serve as proof of an event, and it can be used as training data to improve the anomaly recognition rate.

### Multi-Class problem

**Figure 11:**
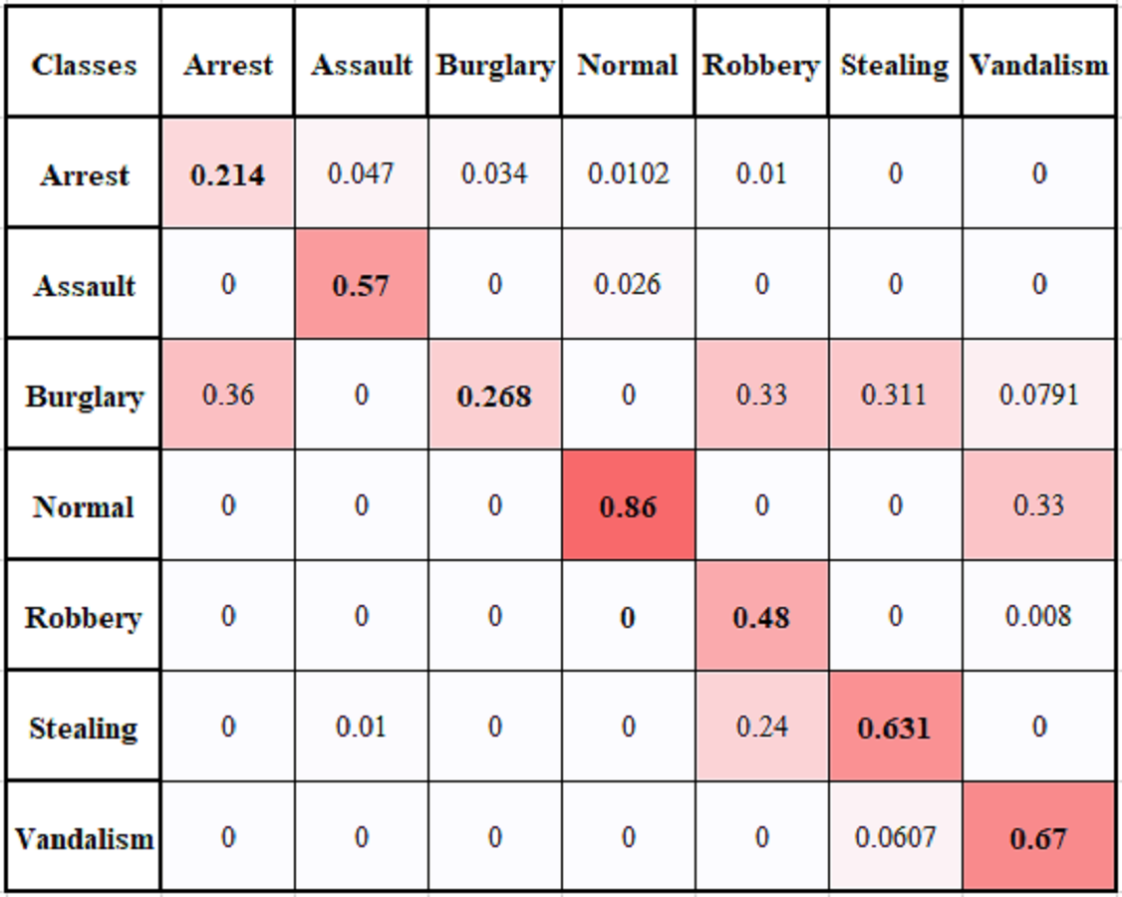
Multiclass confusion matrix of temporal based anomaly recognizer (TAR).

We have performed a multi-class anomaly recognition task on UCF Crime2Local dataset, which has six types of anomalies *e.g.*, Arrest, Assault, Burglary, Robbery, Stealing, and Vandalism. Figure 11 provides the confusion matrix for different classes of UCF Crime2Local, which shows values are comparatively low for classes arrest and burglary. It is because arrest videos usually involve confused events, as arrest act is not clear. Similarly, Burglary act involves clear visibility of event to represent illegal entry. Robbery has also low value because it is sometimes learnt as stealing or assault class. Table 3 provides the comparison of multi-class problem with some baseline methods and our proposed method achieve an average of 52.7% accuracy. The achieved performance is good as it provides a reasonable accuracy and do not require high computational resources. This makes it useful for real-time application, although our focus is to perform two class recognition to identify whether it is an anomaly or not. Overall proposed framework provides suitable accuracy with reduced computational cost.

**Table 3:** Performance of proposed framework (TAR) for multiclass anomaly recognition.

Method	Accuracy
ResNet50	45.20%
MobileNetV2	41.9%
Proposed method	52.70%

## Conclusion

Automated surveillance is popular area which is continuously improving over the time. Such systems are designed to process huge amount of data which requires a lot of resources which is a challenging requirement. In practical scenarios, time, and cost both are critical to handle and hence a system needs a lot of computation time and resources to serve the purpose. Therefore, this research aimed at addressing these problems through providing a resource-efficient, high-performing system for anomaly recognition. Increased numbers of CCTV generates vast amount of unlabelled video data and its labeling is a difficult task. Moreover, large amount of data requires substantial computational resources to process it. This study provides a lightweight and cost-effective approach for anomaly recognition in terms of memory consumption, processing parameters, and computation time that is essential for a low-resource systems, such as CPU-based systems. The proposed framework is based on 2D convolutional architecture (2D CNN), with a spatiotemporal feature extractor which functions as a partial shift that learns and distributes temporal information among its neighborhood frames. MobileNetV2 baseline performs spatial feature extraction, which is then combined with temporal learning to perform anomaly recognition. Our proposed framework works with low latency rate of 12.01 ms which makes it effective for performing online video recognition and handle up to six streams at once. On the UCF Crime dataset, the proposed framework achieves an accuracy of 88% for binary anomaly recognition problem with time complexity of 0.198 s. On the UCF Crime2Local dataset, the proposed framework achieves accuracy of 52.7 percent for a multi-class problem. The model outperforms previous models in terms of computational parameters requiring 2.2 M parameters and 0.564 GFLOPs with MobileNetV2 as the baseline architecture. Moreover, our proposed framework has achieved an increased accuracy of 2.47% on UCF Crime dataset with reduced computational requirement. Overall, it performs well in a lot of aspects and can be used for realtime recognition but it can be further improved. Some limitations of this study are highlighted in below section to consider in future.

## Limitations and Future Directions

We have proposed a resource-efficient anomaly recognition system that effectively performs recognition tasks, but it is not evaluated for object level detection and tracking. Object detection and tracking can significantly improve security surveillance. We believe that with minor modifications to the current model, it could be useful for detection and tracking as well. Our model performs recognition with adequate speed efficiency, but its early response efficiency can be investigated further. It requires validating a model’s ability to perform recognition on a small sample of incoming frames as soon as possible to improve system’s response time. Surveillance systems are designed to perform anomaly recognition as precisely as possible, but there is an additional issue posed by the false positive rate, which can compromise system reliability. To increase the usefulness of our model, we will strive to reduce the false positive rate as much as possible.

## Supplemental Information

10.7717/peerj-cs.1117/supp-1 Supplemental Information 1 Complete Engine with Dataset

## Additional Information and Declarations

### Data Availability

The following information was supplied regarding data availability: The data is available at GitHub: https://github.com/GulshanSaleem/Surveilia. The data is available at Zenodo: Saleem, Gulshan, Bajwa, Usama Ijaz, Raza, Rana Hammad, Alqahtani, Fayez, Tolba, Amr, & Xia, Feng. (2022). Efficient Anomaly Recognition Using Surveillance Videos (V1.1). Zenodo. https://doi.org/10.5281/zenodo.7047216. The UCF Crime dataset was used for training and testing. The video from the UCF Crime dataset is available at: https://www.crcv.ucf.edu/projects/real-world/.
