# Supplementary figures and images for: Efficient anomaly recognition using surveillance videos
Source: PeerJ Comput Sci. 2022 Oct 14;8:e1117. doi: 10.7717/peerj-cs.1117 (PMC9575851; doi:10.7717/peerj-cs.1117)

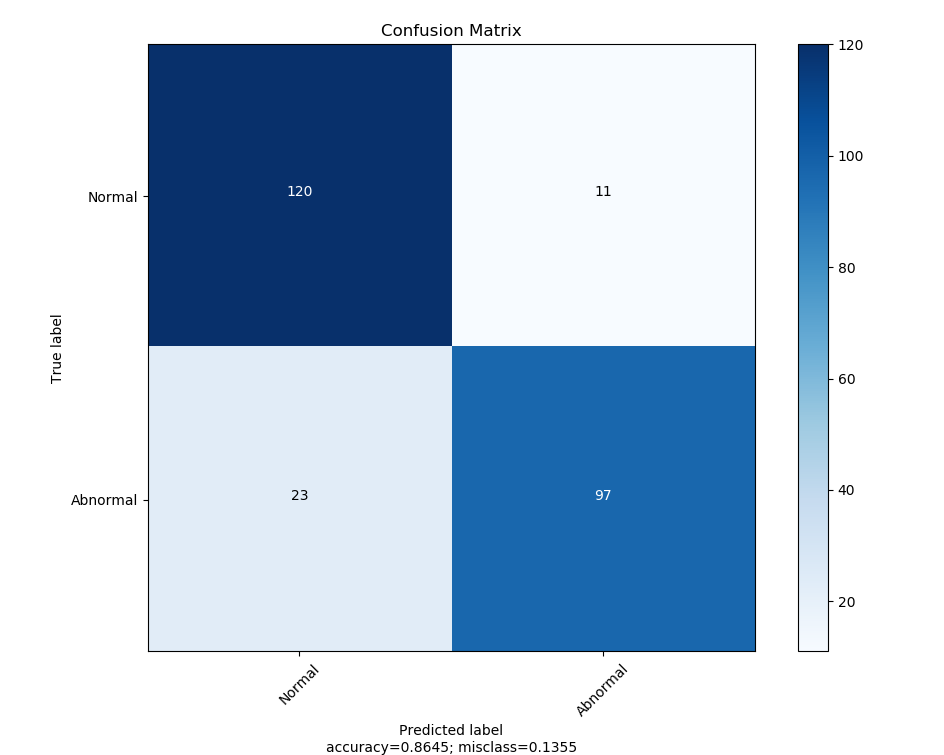

Supplement: Supplemental Information 1 [file peerj-cs-08-1117-s001.zip › cs-72336-Training-and-Core-Infernce-Engine-master/Training-and-Core-Infernce-Engine-master/Confusion Matrix/CF_of_TestData_using_untrimmed_dataset.png]
